# Effects of Synergistic Inhibition on α-glucosidase by Phytoalexins in Soybeans

**DOI:** 10.3390/biom9120828

**Published:** 2019-12-05

**Authors:** Hyeong-U Son, Eun-Kyeong Yoon, Chi-Yeol Yoo, Chul-Hong Park, Myung-Ae Bae, Tae-Ho Kim, Chang Hyung Lee, Ki Won Lee, Hogyun Seo, Kyung-Jin Kim, Sang-Han Lee

**Affiliations:** 1 School of Food Science & Biotechnology, Graduate School, Kyungpook National University, Daegu 41566, Korea; shwcrystal@naver.com (H.-U.S.); sbr9707@naver.com (E.-K.Y.); seeyou0581@hanmail.net (C.-Y.Y.); 2 Pennington Biomedical Research Center, Louisiana State University, Baton Rouge, LA 70808, USA; bbangbbang2@hanmail.net; 3 Korea Research Institute of Chemical Technology, Daejeon 34114, Korea; mbae@krict.re.kr; 4 Biomedical Research Institute, Kyungpook National University Hospital, Daegu 41940, Korea; kimth0929@ynu.ac.kr; 5 Major in Biomodulation, Department of Agricultural Biotechnology and Research Institute for Agriculture and Life Sciences, Seoul National University, Seoul 08826, Korea; changhyungds@naver.com (C.H.L.); kiwon@snu.ac.kr (K.W.L.); 6 School of Life Sciences, KNU Creative BioResearch Group, Institute of Microorganisms, Kyungpook National University, Daegu 41566, Korea; bbndd3@naver.com (H.S.); kkim@knu.ac.kr (K.-J.K.); 7 knu BnC, Daegu 41566, Korea

**Keywords:** α-glucosidase inhibitor, glyceollin, genistein, luteolin, daidzein, phytoalexins, enzyme kinetics, combination index

## Abstract

To determine the mechanism of action of the effects of phytoalexins in soybeans, we analyzed *α*-glucosidase inhibition kinetics using Michaelis–Menten plots and Lineweaver–Burk plots. The results showed that the type of inhibition with glyceollin was competitive, that of genistein was noncompetitive, that of daidzein was uncompetitive, and luteolin showed a mixed mode of action. The *K_i_* values were determined using a Dixon plot as glyceollin, 18.99 μM; genistein, 15.42 μM; luteolin, 16.81 μM; and daidzein, 9.99 μM. Furthermore, potential synergistic effects between glyceollin and the three polyphenols were investigated. A combination of glyceollin and luteolin at a ratio of 3:7 exhibited synergistic effects on *α*-glucosidase inhibition, having a combination index (CI) of 0.64244, according to the CI–isobologram equation. Collectively, these results showed that a combination of glyceollin and luteolin has the potential to inhibit *α*-glucosidase activity via a synergistic mode of inhibition.

## 1. Introduction

Soybeans are an important food resource that has beneficial purposes as “meat in the field” because of its nutritional value [1]. Presently, most Asian countries produce soybeans to use as food ingredients, feed additives, biomaterials, etc. [2]. A high dietary intake of soybeans can reduce the risk of breast cancer and coronary heart disease and exerts anti-diabetic effects by enhancing glucose uptake [3,4,5]. Soybeans contain phytoalexin compounds, such as glyceollin, genistein, luteolin, and daidzein. Phytoalexins include isoflavones which are induced by plant defense mechanisms against soil pathogens, such as bacteria and fungi [6]. Phytoalexins are also produced when soybeans are exposed to various stresses, such as microbes, ultraviolet light, and other physical attacks [7]. Soybean-derived polyphenol compounds are known to exert antioxidant, antitumor, anti-inflammatory, anti-obesity, and anti-atopic effects [8,9,10].

Glyceollins, which are synthesized from daidzein in soybeans infected with fungi, are potent antioxidants that inhibit the production of reactive oxygen species (ROS) [11]. Glyceollins also display anti-inflammatory effects by suppressing the nuclear factor kappa B (NF-κB) signaling pathway [12]. Glyceollins not only exert antitumor effects by inhibiting angiogenesis, but also have antimelanogenic activity by reducing melanin biosynthesis [13,14]. Daidzein is converted into glyceollin in soybeans during fermentation after infection by a fungus such as *Aspergillus sojae*. However, data on the enzymatic effects of glyceollins derived from phytoalexins and studies on how glyceollins and other anti-diabetic agents are involved in the inhibition of *α*-glucosidase are lacking.

The enzyme α-glucosidase (EC 3.2.1.20; alternatively named maltase, glucoinvertase, glucosidosucrase, maltase-glucoamylase, α-glucopyranosidase, glucosido-invertase, α-D-glucosidase, α-glycoside hydrolase, α-1,4-glucosidase, or α-D-glucoside glucohydrolase) catalyzes the exohydrolysis of 1,4-α-glucosidic linkages, and hydrolyzes oligosaccharides rapidly relative to polysaccharides [15]. The enzyme hydrolyzes terminal, non-reducing (1 → 4)-linked α-glucose residues to release a single α-glucose molecule. The substrate selectivity of α-glucosidase is due to the substrate affinity for the active site of the enzyme [16]. In individuals with type 2 diabetes, α-glucosidase induces postprandial hyperglycemia by breaking di-, tri-, and oligosaccharides into monosaccharides, and α-glucosidase inhibitors delay carbohydrate digestion and absorption, thereby attenuating postprandial hyperglycemia [17]. The anti-diabetic potential of α-glucosidase inhibition has been seen with *p*-nitrophenyl α-D-glucopyranoside (pNPG), and there is mounting evidence that various α-glucosidase inhibitors produced by plants such as legumes also exert anti-diabetic effects [18,19,20].

In the present study, we first compared the ability of four polyphenol compounds (glyceollin, genistein, luteolin, and daidzein) derived from soybeans to inhibit α-glucosidase, and then studied the inhibitory mechanism of action by calculating the *K_i_* values of the four polyphenol compounds. We also investigated the synergistic effects between glyceollin and the other three polyphenol compounds. A deeper understanding of the enzyme kinetics and mode of action of glyceollin could help to develop nutraceuticals that prevent diabetes without side effects. Our results indicate that a combination of glyceollin and luteolin has synergistic effects on α-glucosidase inhibition.

## 2. Materials and Methods

### 2.1. Reagents

**Figure 1:**
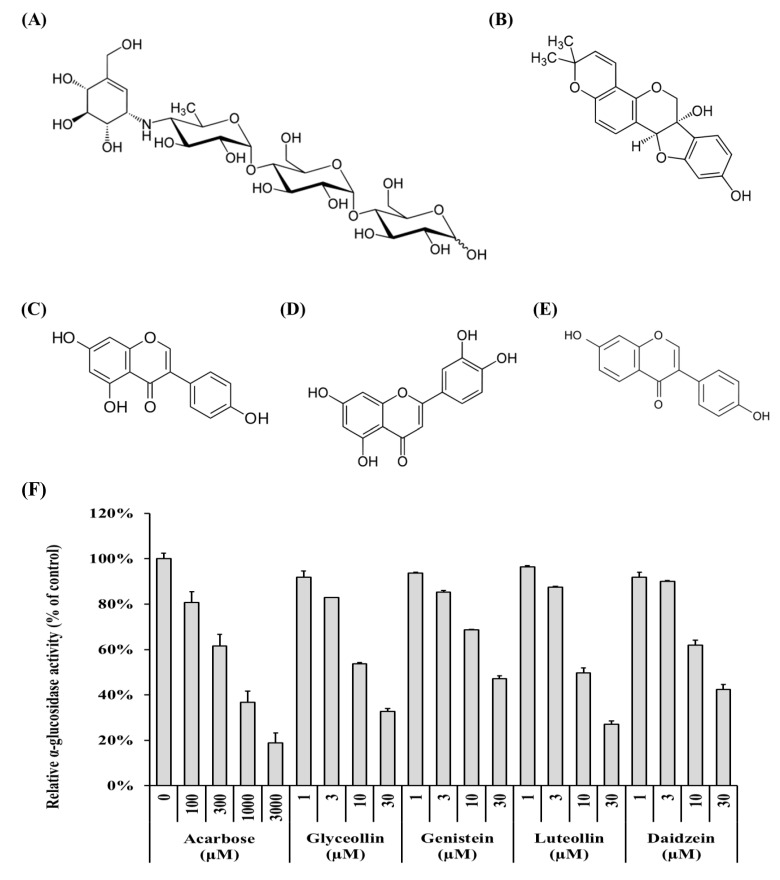
Structures of soybean-derived polyphenol compounds used in this study and their α-glucosidase inhibitory activity. (**A**) Acarbose, a positive control, (**B**) glyceollin, (**C**) genistein, (**D**) luteolin, and (**E**) daidzein. Relative α-glucosidase activity (**F**) was calculated as a relative percentage of the control group. The enzyme activity was calculated as described in Materials and Methods.

α-Glucosidase (from baker’s yeast; #63231-63-0; EC:3.2.1.20), pNPG, acarbose (≥95%), genistein (≥98%), daidzein (≥98%), and luteolin (≥98%) were purchased from Sigma-Aldrich (St, Louis, MO, USA). Glyceollin, which has three isomers, was semi-purified from elicited soybeans, as described previously [21]. All reagents were of analytical grade and prepared at various concentrations by dissolution in dimethyl sulfoxide (DMSO), except for water-soluble acarbose. The structures of the compounds are shown in Figure 1.

### 2.2. Inhibition of α-glucosidase

The activity of α-glucosidase was measured as described previously, with slight modifications [22]. In brief, sodium phosphate buffer (0.1 M) was adjusted to 0.1 N HCl to pH 7.0 with a pH meter (Thermo Fisher Scientific Inc., Waltham, MA, USA). Solutions of pNPG (10 mM) and α-glucosidase (1 U/mL) were solubilized in 0.1 M sodium phosphate buffer (pH 7.0). All reagents were prepared shortly before use and warmed to 37 °C in a water bath (Il-shin Biobase, Pocheon, Korea). Polyphenols (1–30 μM) and acarbose (100–3000 μM, A8980, Sigma) were placed in a 96-well plate. The substrate, pNPG (N1377, Sigma), was added into each well to a final concentration of 1 mM in a total volume of 200 μL, and 100 μL of α-glucosidase (1 U/mL) per well was then added. The absorbance at 405 nm was measured immediately using a spectrophotometer (Victor3 multi-label counter, Wallac, Turku, Finland) at 37 °C and then every 2 min for 40 min. The absorbance values were plotted against time, and the rate (velocity) of product generation (α-glucosidase activity) was calculated from the linear range of the graph.

### 2.3. Enzyme Kinetics for α-glucosidase

The enzyme reaction was performed according to the above reaction conditions with polyphenols at various concentrations (1–30 μM). pNPG (1 mM) was added with polyphenols in 96-well plates, and α-glucosidase (1 U/mL) was added to initiate the enzyme reaction. The absorbances for each concentration of pNPG were then obtained by spectrophotometry (Victor3, PerkinElmer, Waltham, MA, USA). The inhibition modes of the polyphenols were determined using Michaelis–Menten and Lineweaver–Burk equations [23,24]. The inhibition constant *K_i_* is an indication of the potency of an inhibitor and equals the concentration required to produce half-maximal inhibition and can be determined by a Dixon plot [25]. To describe how these agents inhibit α-glucosidase, Lineweaver–Burk equations, in double reciprocal form, are expressed as follows:

$\frac{1}{v}=\;\frac{Km}{Vmax}\;\left(1+\frac{1}{Ki}\right)\times \frac{1}{\left[S\right]}+\frac{1}{Vmax}\;$(Competitive inhibition)
$\frac{1}{v}=\;\frac{Km}{Vmax}\;\times \;\frac{1}{\left[S\right]}\;+\;\frac{1}{Vmax}\left(1+\frac{\left[I\right]}{Ki}\right)\;$(Uncompetitive inhibition)
$\frac{1}{v}=\;\frac{Km}{Vmax}\;\times \left(1\;+\;\frac{\left[I\right]}{Ki}\right)\;\times \;\frac{1}{\left[S\right]}\;+\;\frac{1}{Vmax}\left(1+\frac{\left[I\right]}{Ki}\right)\;$(Noncompetitive inhibition)
$\frac{1}{v}=\;\frac{Ks}{Vmax}\left(1+\frac{\left[I\right]}{Ki}\right)\times \frac{1}{\left[S\right]}+\frac{1}{Vmax}\left(1+\frac{\left[I\right]}{\alpha Ki}\right)\;$(Mixed inhibition)

where *v* is the enzyme reaction rate in the absence and presence of inhibitors or polyphenol compounds; *V_max_* and [*S*] are the maximum reaction velocity and the substrate concentration, respectively; and *K_m_* and *K_s_* are the Michaelis–Menten and dissociation constants for the affinity of the substrate, respectively. The *α* symbol is the ratio of the uncompetitive inhibition constant to the competitive inhibition constant and has a value of 1 for noncompetitive inhibition. The *αK_i_* value is the inhibitor constant when the inhibitor (I) occupies the enzyme–substrate (ES) complex [25].

### 2.4. Docking Studies

To investigate the modes of α-glucosidase inhibition for individual polyphenols, docking calculations were performed by Autodock Vina software, a program that simulates molecular docking and virtual screening, which is improved compared to the average accuracy of the binding mode predictions of AutoDock 4.0 [26]. Three-dimensional coordinates of α-glucosidase used as the input structure were prepared by modeling using the SWISS-MODEL server [27]. The protein modeling used the structure of isomaltase from *Saccharomyces cerevisiae* (PDB code 3AJ7) as a template, having 73% amino acid identity with the commercial α-glucosidase, MAL12. For the docking calculation, the generation of the *pdbqt* files and determination of the grid box size were done with AutoDock Tools, Version 1.5.4 software (http://mgltools.scripps.edu/). Default parameters, except the exhaustiveness option, were used as described in the AutoDock Vina manual. The side chain of residues (Asp68, Tyr71, His111, Phe151, Phe177, Arg212, Asp214, Thr215, His239, Glu276, His279, Phe300, Arg312, His348, Asp349, and Arg439) were allowed to rotate and an active site defined by a grid box was centered at x = 17.471, y = −7.312, and z = 21.611 with size of 46 × 36 × 48 Å (0.375 spacing). Nine output poses were generated and evaluated by their calculated free energy of binding. The best theoretical binding mode of each inhibitor was selected by its ΔG_bind_ score.

### 2.5. Synergistic Effects on α-glucosidase Inhibition

Synergistic effects on α-glucosidase inhibition were measured using the same methods as above. A designated concentration (0.5–2.0 mM) of pNPG, together with a combination of two polyphenols, was added to a 96-well plate, and the enzyme reaction was initiated by the addition of α-glucosidase (1 U/mL). We calculated the IC_50_ values of each polyphenol and the combinations, and we used the statistical differences of these values to assess synergistic effects.

### 2.6. Determination of the Combination Index (CI)

CI values were calculated by the method of Chou [28], which offers a theoretical basis for the CI–isobologram equation that permits the quantitative determination of compound interactions. Based on the algorithm, computer software has been established to simulate synergism and antagonism at any ratio. In brief, the derived CI equation for two compounds is:

CI = (D)1/(Dx)1 + (D)2/(Dx)2 = (D)1/(Dm)1[fa/(1 − fa)]^1/m1^ + (D)2/(Dm)2[fa/(1 − fa)]^1/m2^

where (Dx)1 for (D)1 “alone” that inhibits a system x%, and (Dx)2 for (D)2 “alone” that inhibits a system x% whereas in the numerator, (D)1 + (D)2, “in combination” also inhibit x%. Note that the denominators of the last two terms are expressed as median effects. The CI value quantitatively defines synergism (CI < 1), an additive effect (CI = 1), and antagonism (CI > 1).

In single drug dynamics, the equation is defined as fa/fu = [D/Dm]^m^, where fa is the affected fraction of the system and fu is the unaffected fraction (fu = 1 − fa); D is the dose required to produce fa; Dm is the dose required to produce median effects, such as ED_50_ or IC_50_; and m is sigmoidicity (shape).

### 2.7. Statistical Analysis

The results are presented as the mean ± standard deviation (SD) of experiments performed in triplicate. Statistical differences were determined by Tukey’s one-way ANOVA using IBM SPSS software (Armonk, NY, USA). A difference was considered significant at *p* < 0.05.

## 3. Results

### 3.1. Potential of Phytoalexins Derived from Soybeans to Inhibit α-Glucosidase

The ability of polyphenol compounds derived from soybeans to inhibit α-glucosidase (Figure 1B–E) was measured to identify which compound is most effective. Acarbose (Figure 1A) is an anti-diabetic drug [19] and was used as a positive control in the α-glucosidase inhibition assays [29]. All compounds tested reduce α-glucosidase activity in a concentration-dependent manner (Figure 1F, grey columns). The half-maximal inhibitory concentrations (IC_50_) of acarbose, glyceollin, genistein, luteolin, and daidzein were 530.50 ± 100.13 μM, 13.22 ± 2.31 μM, 23.66 ± 3.54 μM, 11.94 ± 1.63 μM, and 20.16 ± 6.17 μM, respectively. These results show that the four soybean-derived polyphenol compounds exert α-glucosidase inhibitory activity that is more than 20 times higher than that of acarbose.

### 3.2. Determination of Inhibition Modes and K_i_ Values on Polyphenols Derived from Soybeans on α-glucosidase

**Figure 2:**
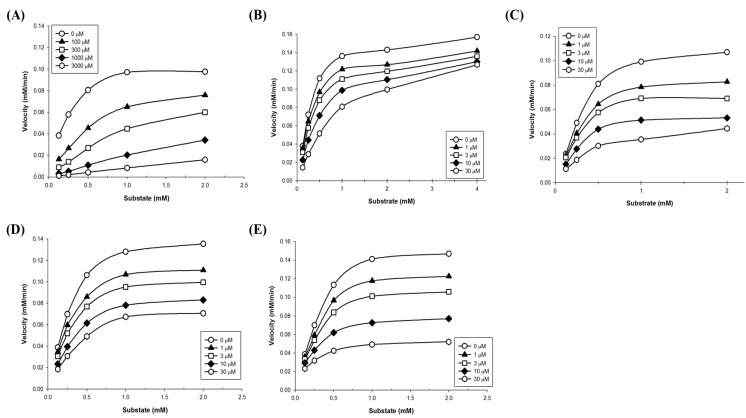
Non-linear regression analysis of α-glucosidase. The inhibitors were applied at the indicated concentrations: (**A**) Acarbose, (**B**) glyceollin, (**C**) genistein, (**D**) luteolin, and (**E**) daidzein. A mixture of 100 μL of each concentration of *p*-nitrophenyl α-D-glucopyranoside (pNPG) was added to 96-well plates that contained polyphenols and treated with α-glucosidase to initiate the enzyme reaction. The Lineweaver–Burk plots show the enzyme kinetics of the four kinds of classical inhibition modes. Each plot represents the results from three independent experiments.

**Figure 3:**
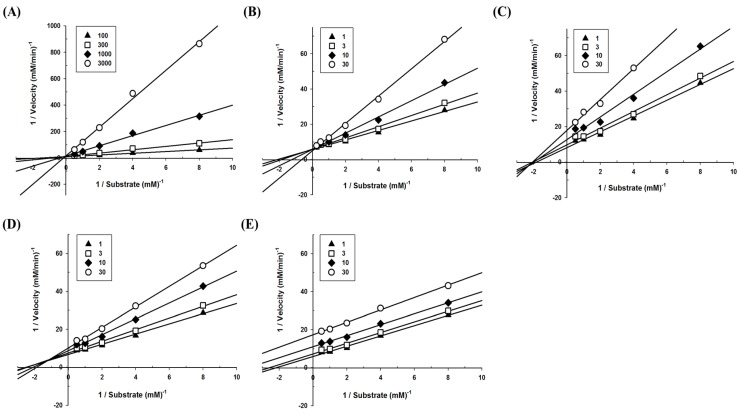
Modes of α-glucosidase inhibition for polyphenols derived from soybeans. To calculate the mode of action, Lineweaver–Burk plots were constructed: (**A**) Acarbose (closed triangle, 100 μM; open square, 300 μM; closed diamond, 1000 μM; open circle, 3000 μM), (**B**) glyceollin (closed triangle, 1 μM; open square, 3 μM; closed diamond, 10 μM; open circle, 30 μM), (**C**) genistein (closed triangle, 1 μM; open square, 3 μM; closed diamond, 10 μM; open circle, 30 μM), (**D**) luteolin (closed triangle, 1 μM; open square, 3 μM; closed diamond, 10 μM; open circle, 30 μM), and (**E**) daidzein (closed triangle, 1 μM; open square, 3 μM; closed diamond, 10 μM; open circle, 30 μM).

To define the mode of inhibition of the polyphenols, enzyme kinetics were performed with designated concentrations of pNPG. The maximum velocity (V_max_) for α-glucosidase was calculated from the nonlinear regression curve with five different concentrations of substrate (Figure 2). To determine the inhibition modes, Lineweaver–Burk plots were constructed for each of the inhibition modes, and the most suitable mode was selected using SigmaPlot 10.0 software. The results reveal that the mode of acarbose and glyceollin inhibition is competitive, while that of genistein is noncompetitive. Luteolin shows mixed inhibition modes, and the mode of daidzein inhibition is uncompetitive (Figure 3).

Meanwhile, the inhibitor constant *K_i_* indicates the potency of an inhibitor and is expressed as the concentration required to produce half-maximal inhibition and is determined by a Dixon plot [25]. The initial slope *v* was determined for each concentration of each polyphenol. The reciprocal velocity (1/*v*) versus the substrate concentration (for 0.25, 0.5, 1, and 2 mM pNPG) was plotted. A single regression line for each concentration of the substrate was obtained, and *K_i_* was calculated from the intersection of the four lines. The *K_i_* values were determined by GraphPad Prism 6.0 software as 45.88 ± 3.75 μM, 18.99 ± 4.45 μM, 15.42 ± 2.48 μM, and 16.81 ± 9.60 μM, for acarbose, glyceollin, genistein, and luteolin, respectively (Table 1). It is not possible to calculate *K_i_* in the case of uncompetitive inhibition because the four lines do not intersect. Therefore, for daidzein, the *αK_i_* value was calculated; the *αK_i_* constant is the inhibitor constant when an inhibitor occupies the enzyme–substrate complex. The *αK_i_* value of daidzein was calculated as 9.99 ± 1.24 μM.

### 3.3. Structural Bases for the Modes of Inhibition

**Figure 4:**
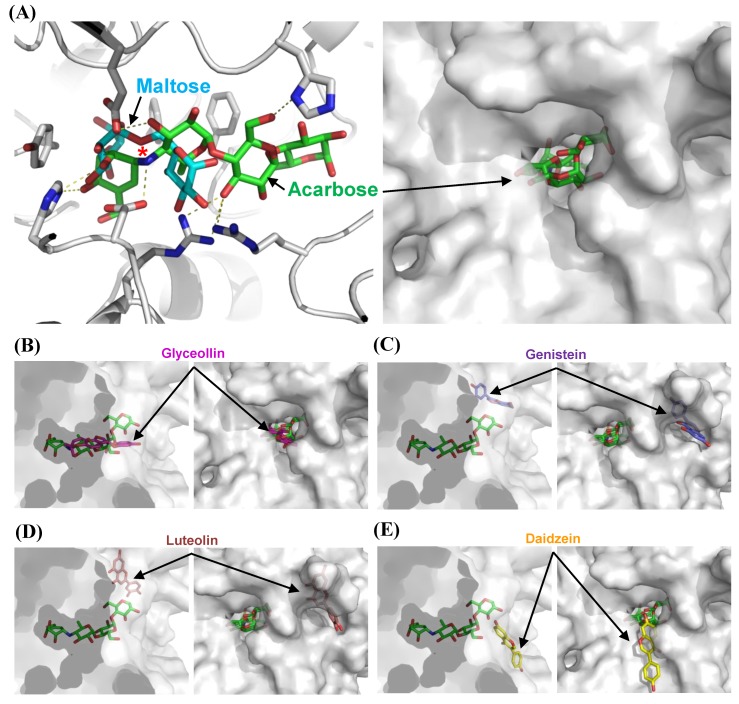
Molecular docking studies on the modes of α-glucosidase inhibition. The model of MAL12, a structure of α-glucosidase, is shown as a cartoon or surface diagram in white. Isomaltose, acarbose, glyceollin, genistein, luteolin, and daidzein are shown as stick diagrams. (**A**) The inhibitory mode of acarbose. The isomaltose molecule was prepared by superposing the model of MAL12 with the structure of isomaltase (PDB code 3AXH) and is shown in cyan. Residues involved in substrate binding are displayed as stick models. Yellow dashed lines indicate hydrogen bond interactions. The glucosidic bonds to be hydrolyzed are indicated by a star. (**B**–**E**) The inhibitory modes of polyphenols. Glyceollin, genistein, luteolin, and daidzein are shown as stick diagrams in magenta, purple, yellow, and brown, respectively.

To understand the modes of inhibition at a molecular level, we performed docking studies using the structure-guided model of α-glucosidase. Docking models of acarbose, glyceollin, genistein, luteolin, and daidzein were generated, and the best models of each inhibitor were chosen (Figure 4A–E). First, we compared the docking model of acarbose with the homologous structure complexed with maltose. The superimposition reveals that acarbose is docked into the pocket in which the substrate isomaltose binds experimentally, and a nitrogen atom is located near the glycosidic bond of the substrate to block hydrolysis (Figure 4A). The result explains why the acarbose competitively binds to α-glucosidase and inhibits the enzyme. We then used the acarbose model as an indicator of the substrate-binding site, onto which the other docking models were superimposed.

The α-glucosidase is applied to the docking model of glyceollin, which is a pterocarpan that is structurally different from other isoflavones and flavone, with its substrate-binding pocket (Figure 4B). The theoretical binding affinity (ΔG_bind_) is −10.3 kcal/mol. This structural model illustrates the competitive inhibition of glyceollin. Although the polyphenols tested are similar in structure, they dock in the active site pocket with different orientations. Interestingly, the model with daidzein shows that the substrate covers the entrance of the pocket with a ΔG_bind_ of −7.6 kcal/mol (Figure 4E). Although an uncompetitive inhibitor binds only to the enzyme–substrate complex, the relative affinity of the binding compared to that of glyceollin might be lower. Consequently, the model shows the binding cavity of the uncompetitive inhibitor and might explain the rare uncompetitive inhibition mode as described above. Luteolin and genistein are proposed to be mixed and noncompetitive inhibitors, respectively. A mixed inhibitor binds both the apoenzyme and the enzyme–substrate complex, and it binds a different site than the substrate-binding pocket, altering the active-site configuration and the enzymatic turnover. Noncompetitive inhibition is a special case of mixed inhibition under an unchanged *K_m_*. Surprisingly, the docking models show a different cavity that harbors luteolin and genistein with ΔG_bind_ values of −8.4 and −8.7 kcal/mol, respectively, intimating that noncompetitive or mixed inhibition might occur by altering the catalytic cleft upon attachment (Figure 4C,D).

### 3.4. Combined Effects of Glyceollin Plus Luteolin on α-glucosidase Inhibition

We studied the α-glucosidase inhibitory effect of pairs of polyphenols by mixing glyceollin with luteolin, genistein, or daidzein. As shown in Table 2, we obtained IC_50_ values for each polyphenol singly and the combination treatments on α-glucosidase inhibition at each concentration of pNPG. Interestingly, luteolin shows a combined effect on α-glucosidase inhibition when combined with glyceollin. However, neither genistein nor daidzein shows a combined effect (Table 2, bold characters).

**Figure 5:**
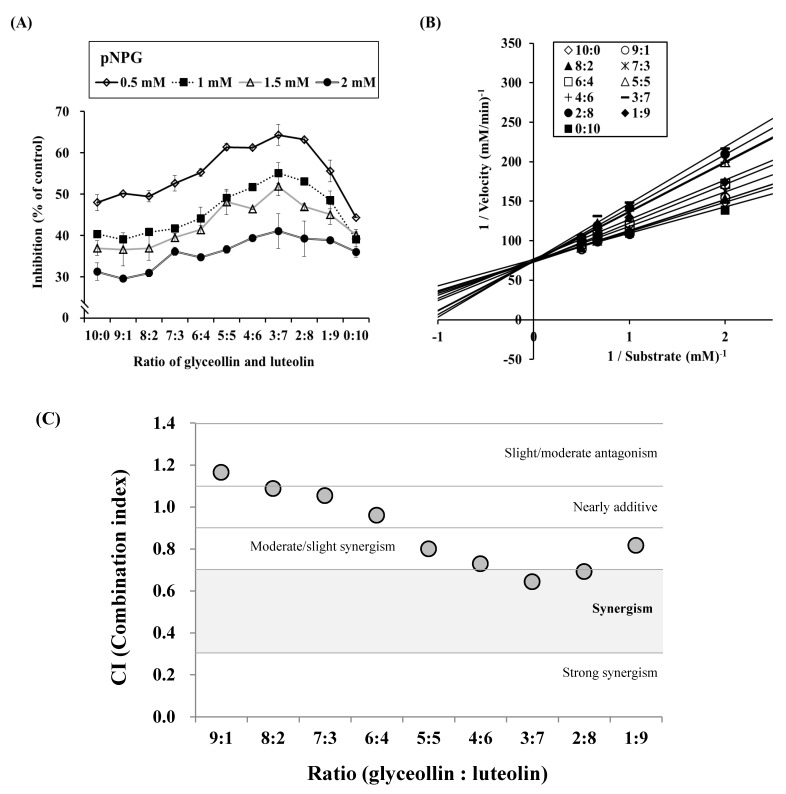
Synergistic effect of glyceollin plus luteolin on α-glucosidase inhibition. Glyceollin and luteolin were each prepared as 2 μM solutions and mixed in ratios ranging from 0:10 to 10:0. Therefore, a 5:5 ratio indicates mixture of 1 μM glyceollin plus 1 μM luteolin. (**A**) The pattern of α-glucosidase inhibition according to four concentrations of substrate (0.5, 1, 1.5, and 2 mM). (**B**) Lineweaver–Burk plots of the effects of glyceollin plus luteolin mixtures at various ratios. (**C**) Combination index (CI) plot of various ratios of glyceollin and luteolin. The CI–isobologram equation permits quantitative determination of compound interactions and CI < 1, CI = 1, and CI > 1 show synergism, an additive effect, and antagonism, respectively.

We further investigated these combined effects of glyceollin and luteolin by making mixtures in ratios ranging from 0:10 to 10:0 and then testing their inhibition of *α*-glucosidase. The results show that a 3:7 ratio of glyceollin to luteolin causes the highest inhibition of *α*-glucosidase. We then plotted the kinetic modes of action in Figure 5B. Surprisingly, the data show that the mode of action is competitive inhibition. These results indicate that a combination of competitive and mixed inhibition modes might exert more potent inhibition on α-glucosidase activity, whereas a combination of competitive and uncompetitive or noncompetitive modes are less potent.

### 3.5. Confirmation of Synergism by CI Equation with Glyceollin Plus Luteolin

To scrutinize whether the inhibition mode of the combination of glyceollin and luteolin is synergistic, we calculated CI values for experimental data by the method of Chou [28]. Four data points for glyceollin or luteolin (1, 3, 10, and 30 μM) were entered into the equation, and the resulting *K_m_* values were 13.6215 and 12.2697, respectively. As shown in Figure 5C, the ratios of glyceollin to luteolin between 9:1 to 7:3 show CI values >1.0. The ratios of glyceollin to luteolin between 6:4 to 1:9 yield CI values <1.0. Interestingly, the 3:7 ratio produces the lowest CI value, suggesting that this ratio ranks as having the most synergistic effect on *α*-glucosidase inhibition.

## 4. Discussion

In a study of α-glucosidase related to postprandial hyperglycemia in type 2 diabetes, glyceollin showed an effect similar to three known polyphenols (genistein, luteolin, and daidzein; Table 1). To the best of our knowledge, this is the first time that glyceollin was revealed to be a competitive inhibitor of α-glucosidase (Figure 3B). Competitive and mixed inhibitors exert their effects by combining with free enzyme to prevent substrate binding, thus producing an enzyme–inhibitor (EI) complex. By contrast, noncompetitive or uncompetitive inhibitors cannot directly interrupt the binding of the enzyme to its substrate. We hypothesized that a synergistic effect would occur between competitive and noncompetitive (or uncompetitive) inhibitors because the binding sites of the inhibitors are different. However, combinations of competitive and noncompetitive (or uncompetitive) inhibitors decreased the inhibition activity (Table 2). Interestingly, glyceollin (a competitive inhibitor) and luteolin (a mixed inhibitor) displayed a significant synergistic effect, with decreased IC_50_ values at all concentrations of the substrate (Table 2).

General biological mechanisms are not limited to the reaction of a single compound. Therefore, complicated synergistic mechanisms are likely to be involved in many biological events in other investigations of mechanisms of α-glucosidase inhibition [30]. Liu et al. demonstrated that a combination of inhibitors improves α-glucosidase inhibition [31]. They selected two typical xanthone derivatives (1,3,7-trihydroxyxanthone and 1,3-dihydroxybenzoxanthone) and observed their synergistic effect. In their study, 2 μM of 1,3,7-trihydroxyxanthone exhibited approximately 15% inhibition, while 2 μM of 1,3-dihydroxybenzoxanthone exhibited approximately 10% inhibition. Interestingly, the synergistic effect of combining the two inhibitors at a 3:7 ratio produced a maximal inhibition of 40% [31]. Another study on α-glucosidase demonstrated genistein synergistic inhibition when combined with metal ions such as copper and zinc [30]. Therefore, we hypothesized that a mixture of two polyphenols would exert synergistic inhibition against α-glucosidase. Indeed, glyceollin showed a synergistic effect with luteolin on α-glucosidase inhibition (Table 2). When acarbose, which is a competitive inhibitor like glyceollin, was used in combination with luteolin or glyceollin, no synergistic effects were observed. Combined treatment with genistein and daidzein also produced no synergistic effects.

It was shown that dose and effect are interchangeable via defined parameters derived from the unified theory for the Michaelis–Menten equation, Hill equation, Henderson–Hasselbalch equation, and Scatchard equation. These equations provide the theoretical basis for the combination index (CI)–isobologram equation that allows quantitative determination of compound interactions, where CI < 1, CI = 1, and CI > 1 denote synergism, an additive effect, and antagonism [28], respectively. In this study, by applying a unique and effective systemic method, we disclosed that a combination of glyceollin (a competitive inhibitor) and luteolin (a mixed inhibitor) displayed a significant synergistic effect, with a decreased maximal CI value (0.64244) at a 3:7 ratio of glyceollin to luteolin, theoretically (Figure 5C). This theoretical approach can be applied to various studies in biomolecular sciences, from how to effectively evaluate an extract by food ingredient(s), to how to beneficially use multiple compounds, or modalities, in combination therapies for the development of nutraceuticals or functional biomolecules.

To verify whether the combination of glyceollin and luteolin affects the mode of α-glucosidase inhibition, it was necessary to analyze the affinity of each of the polyphenol compounds. When soybeans are infected with various elicitors, polyphenol compounds are naturally produced. Such polyphenol compounds are called phytoalexins, although the identification of the polyphenols is still needed. This study investigated the synergistic potency of two polyphenols by comparing their *K_i_* values; however, we do not know how the structure of the enzyme is changed by the binding of inhibitors. Therefore, it would be useful to perform molecular docking simulation studies and protein structure analyses, including X-ray crystallography and nuclear magnetic resonance (NMR), to determine the mode of synergistic inhibition of soybean-derived polyphenols, which will shed light on their inhibitory mechanisms.

## 5. Conclusions

By analyzing enzyme inhibition kinetics using Michaelis–Menten plots and Lineweaver–Burk plots, we found that inhibition mode of glyceollin was competitive inhibition, that of genistein was noncompetitive, that of daidzein was uncompetitive, and luteolin showed a mixed mode of action. These results indicate that glyceollin, genistein, luteolin, and daidzein could be promising α-glucosidase inhibitors for anti-diabetic approaches. A combination of glyceollin and luteolin had synergistic effects on *α*-glucosidase inhibition, showing that a combination of glyceollin and luteolin has the potential to inhibit *α*-glucosidase activity via a synergistic mode of action. The inhibition of α-glucosidase by polyphenol compounds from soybeans is not so high when the soybeans are not elicited by *Aspergillus*. However, the glyceollin produced by fermentation in response to *Aspergillus* or other fungi is thought to rapidly enhance the antidiabetic effect. Collectively, we believe that fermentation of soybeans induces various phytoalexins (e.g., glyceollins in soybeans); therefore, the intake of fermented soybeans in foods such as soy sauce, soy paste, Koji, and Natto might be useful to synergistically prevent and/or control type 2 diabetes mellitus.

## Figures and Tables

**Table 1:** *K_i_* values of polyphenols derived from soybeans for α-glucosidase inhibition. *K_i_* values of acarbose, glyceollin, genistein, luteolin, and daidzein were calculated using GraphPad Prism 6.0 software. For daidzein, an α*K_i_* value is provided instead of a *K_i_* value.

Inhibitor	Mode of Inhibition	*K_m_* (μm)	*K_i_* (μm)
Acarbose	Competitive	0.21 ± 0.02	45.88 ± 3.75
Glyceollin	Competitive	0.24 ± 0.03	18.99 ± 4.45
Genistein	Noncompetitive	0.36 ± 0.06	15.42 ± 2.48
Luteolin	Mixed	0.30 ± 0.06	16.81 ± 9.60
Daidzein	Uncompetitive	0.40 ± 0.05	9.99 ± 1.24

**Table 2:** Synergistic effects of glyceollin in combination with three polyphenols derived from soybeans on α-glucosidase inhibition.

IC_50_	Mode of Inhibition	Substrate (PNPG, mM)				Remark
		0.125	0.25	0.5	1	
Glyceollin (μM)	Competitive	16.57 ± 3.25	16.57 ± 0.15	19.11 ± 0.96	33.23 ± 3.97	Synergistic
Luteolin (μM)	Mixed	18.63 ± 1.20	14.82 ± 0.01	15.35 ± 1.99	19.52 ± 1.59	
Glyceollin + Luteolin (μM)		6.31 ± 0.59 *^,#^	6.78 ± 0.18 *^,#^	9.00 ± 0.40 *^,#^	14.16 ± 0.35 *	
Glyceollin (μM)	Competitive	16.57 ± 3.25	16.57 ± 0.15	19.11 ± 0.96	33.23 ± 3.97	Non-synergistic
Genistein (μM)	Noncompetitive	40.47 ± 4.99	15.42 ± 0.33	12.00 ± 0.31	9.10 ± 0.61	
Glyceollin + Genistein (μM)		32.47 ± 1.48	33.73 ± 1.51	20.67 ± 0.44	21.81 ± 0.58	
Glyceollin (μM)	Competitive	16.57 ± 3.25	16.57 ± 0.15	19.11 ± 0.96	33.23 ± 3.97	Non-synergistic
Daidzein (μM)	Uncompetitive	59.16 ± 6.10	27.56 ± 2.93	23.19 ± 1.04	20.53 ± 1.82	
Glyceollin + Daidzein (μM)		33.69 ± 0.65	22.78 ± 1.20	16.05 ± 0.51	19.04 ± 0.08	

* *p* < 0.05: Combination treatment is significantly different from glyceollin alone. *^#^ p* < 0.05: Combination treatment is significantly different from luteolin alone.

## References

[1] Hu C., Wong W.T., Wu R., Lai W.F. (2019). Biochemistry and use of soybean isoflavones in functional food development. Crit. Rev. Food Sci. Nutr..

[2] Rizzo G., Baroni L. (2018). Soy, soy foods and their role in vegetarian diets. Nutrients.

[3] Nagata C., Mizoue T., Tanaka K., Tsuji I., Tamakoshi A., Matsuo K., Wakai K., Inoue M., Tsugane S., Sasazuki S. (2014). Soy intake and breast cancer risk: An evaluation based on a systematic review of epidemiologic evidence among the Japanese population. Jpn. J. Clin. Oncol..

[4] Roblet C., Doyen A., Amiot J., Pilon G., Marette A., Bazinet L. (2014). Enhancement of glucose uptake in muscular cell by soybean charged peptides isolated by electrodialysis with ultrafiltration membranes (EDUF): Activation of the AMPK pathway. Food Chem..

[5] Yu D., Zhang X., Xiang Y.B., Yang G., Li H., Fazio S., Linton M., Cai Q., Zheng W., Gao Y.T. (2014). Association of soy food intake with risk and biomarkers of coronary heart disease in Chinese men. Int. J. Cardiol..

[6] Boué S.M., Isakova I.A., Burow M.E., Cao H., Bhatnagar D., Sarver J.G., Shinde K.V., Erhardt P.W., Heiman M.L. (2012). Glyceollins, soy isoflavone phytoalexins, improve oral glucose disposal by stimulating glucose uptake. J. Agric. Food Chem..

[7] Feng S., Song L., Lee Y.K., Huang D. (2010). The effects of fungal stress on the antioxidant contents of black soybeans under germination. J. Agric. Food Chem..

[8] Choi M.S., Jung U.J., Yeo J., Kim M.J., Lee M.K. (2008). Genistein and daidzein prevent diabetes onset by elevating insulin level and altering hepatic gluconeogenic and lipogenic enzyme activities in non-obese diabetic (NOD) mice. Diabetes Metab. Res. Rev..

[9] Scuro L.S., Simioni P.U., Grabriel D.L., Saviani E.E., Modolo L.V., Tamashiro W.M., Salgado I. (2004). Suppression of nitric oxide production in mouse macrophages by soybean flavonoids accumulated in response to nitroprusside and fungal elicitation. BMC Biochem..

[10] Palacios-Gonzalez B., Flores-Galicia I., Noriega L.G., Alemán-Escondrillas G., Zariñan T., Ulloa-Aguirre A., Torres N., Tovar A.R. (2014). Genistein stimulates fatty acid oxidation in a leptin receptor-independent manner through the JAK2-mediated phosphorylation and activation of AMPK in skeletal muscle. Biochim. Biophys. Acta.

[11] Yoon E.K., Kim H.K., Cui S., Kim Y.H., Lee S.-H. (2012). Soybean glyceollins mitigate inducible nitric oxide synthase and cyclooxygenase-2 expression levels via suppression of the NF-κB signaling pathway in RAW 264.7 cells. Int. J. Mol. Med..

[12] Lee S.-H., Lee J., Jung M.H., Lee Y.M. (2013). Glyceollins, a novel class of soy phytoalexins, inhibit angiogenesis by blocking the VEGF and bFGF signaling pathways. Mol. Nutr. Food Res..

[13] Kim H.J., Suh H.J., Kim J.H., Park S., Joo Y.C., Kim J.S. (2010). Antioxidant activity of glyceollins derived from soybean elicited with *Aspergillus sojae*. J. Agric. Food Chem..

[14] Lee Y.S., Kim H.K., Lee K.J., Jeon H.W., Cui S., Lee Y.M., Moon B.J., Kim Y.H., Lee Y.S. (2010). Inhibitory effect of glyceollin isolated from soybean against melanogenesis in B16 melanoma cells. BMB Rep..

[15] Chiba S. (1997). Molecular mechanism in α-glucosidase and glucoamylase. Biosci. Biotechnol. Biochem..

[16] Larner J., Lardy H., Myrback K. (1960). Other glucosidases. The Enzymes 4.

[17] Castro-Acosta M.L., Lenihan-Geels G.N., Corpe C.P., Hall W.L. (2016). Berries and anthocyanins: Promising functional food ingredients with postprandial glycaemia-lowering effects. Proc. Nutr. Soc..

[18] Cao H., Ou J., Chen L., Zhang Y., Szkudelski T., Delmas D., Daglia M., Xiao J. (2019). Dietary polyphenols and type 2 diabetes: Human Study and Clinical Trial. Crit. Rev. Food Sci. Nutr..

[19] Alam M.B., An H., Ra J.S., Lim J.Y., Lee S.H., Yoo C.Y., Lee S.-H. (2018). Gossypol from cottonseeds ameliorates glucose uptake by mimicking insulin signaling and improves glucose homeostasis in mice with streptozotocin-induced diabetes. Oxidative Med. Cell. Longev..

[20] Proença C., Freitas M., Ribeiro D., Oliveira E.F.T., Sousa J.L.C., Tomé S.M., Ramos M.J., Silva A.M.S., Fernandes P.A. (2017). α-Glucosidase inhibition by flavonoids: An in vitro and in silico structure-activity relationship study. J. Enzym. Inhib. Med. Chem..

[21] Yoon E.K., Jeong Y.T., Li X., Cui S., Park D.C., Kim Y.H., Kim Y.D., Chang H.W., Lee S.-H., Hwang S.L. (2013). Glyceollin improves endoplasmic reticulum stress-induced insulin resistance through CaMKK-AMPK pathway in L6 myotubes. J. Nutr. Biochem..

[22] Lee D.S., Lee S.-H. (2001). Genistein, a soy isoflavone, is a potent alpha-glucosidase inhibitor. FEBS Lett..

[23] Kazeem M.I., Adamson J.O., Ogunwande I.A. (2013). Modes of inhibition of α-amylase and α-glucosidase by aqueous extract of *Morinda lucida* Benth leaf. Biomed. Res. Int..

[24] Adisakwattana S., Chantarasinlapin P., Thammarat H., Yibchok-Anun S. (2010). A series of cinnamic acid derivatives and their inhibitory activity on intestinal alpha-glucosidase. J. Enzym. Inhib. Med. Chem..

[25] Thompson W.J., Appleman M.M. (1971). Multiple cyclic nucleotide phosphodiesterase activities from rat brain. Biochemistry.

[26] Trott O., Olson A.J. (2010). AutoDock Vina: Improving the speed and accuracy of docking with a new scoring function, efficient optimization, and multithreading. J. Comput. Chem..

[27] Biasini M., Bienert S., Waterhouse A., Arnold K., Studer G., Schmidt T., Kiefer F., Gallo-Cassarino T., Bertoni M., Bordoli L. (2014). SWISS-MODEL: Modelling protein tertiary and quaternary structure using evolutionary information. Nucleic Acids Res..

[28] Chou T.-C. (2006). Theoretical basis, experiemntal design, and computerized simulation of synergism and antagonism in drug combination studies. Pharm. Rev..

[29] Chiasson J.L., Josse R.G., Gomis R., Hanefeld M., Karasik A., Laakso M. (2002). Acarbose for prevention of type 2 diabetes mellitus: The STOP-NIDDM randomised trial. Lancet.

[30] Wang Y., Ma L., Li Z., Du Z., Liu Z., Qin J., Wang X., Huang Z., Gu L., Chen A.S. (2004). Synergetic inhibition of metal ions and genistein on α-glucosidase. FEBS Lett..

[31] Liu Y., Ma L., Chen W.H., Park H., Ke Z., Wang B. (2013). Binding mechanism and synergetic effects of xanthone derivatives as noncompetitive alpha-glucosidase inhibitors: A theoretical and experimental study. J. Phys. Chem. B.

